# Educational Inequalities in Hospital Use Among Older Adults in England, 2004‐2015

**DOI:** 10.1111/1468-0009.12479

**Published:** 2020-10-06

**Authors:** GEORGE STOYE, BEN ZARANKO, MARTIN SHIPLEY, MARTIN MCKEE, ERIC J. BRUNNER

**Affiliations:** ^1^ Institute for Fiscal Studies and Department of Economics University College London; ^2^ University College London Institute of Epidemiology and Health Care; ^3^ London School of Hygiene and Tropical Medicine

**Keywords:** Health care inequalities, universal health care, educational inequalities, hospital utilization, health care funding, National Health Service

## Abstract

**Policy Points**

- US policymakers considering proposals to expand public health care (such as “Medicare for all”) as a means of reducing inequalities in health care access and use could learn from the experiences of nations where well‐funded universal health care systems are already in place.
- In England, which has a publicly funded universal health care system, the use of core inpatient services by adults 65 years and older is equal across groups defined by education level, after controlling for health status. However, variation among these groups in the use of outpatient and emergency department care developed between 2010 and 2015, a period of relative financial austerity.
- Based on England's experience, introducing universal health care in the United States seems likely to reduce, but not entirely eliminate, inequalities in health care use across different population groups.

**Context:**

Expanding access to health care is once again high on the US political agenda, as is concern about those who are being “left behind.” But is universal health care that is largely free at the point of use sufficient to eliminate inequalities in health care use? To explore this question, we studied variation in the use of hospital care among education‐level‐defined groups of older adults in England, before and after controlling for differences in health status. In England, the National Health Service (NHS) provides health care free to all, but the growth rate for NHS funding has slowed markedly since 2010 during a widespread austerity program, potentially increasing inequalities in access and use.

**Methods:**

Novel linkage of data from six waves (2004‐2015) of the English Longitudinal Study of Ageing (ELSA) with participants’ hospital records (Hospital Episode Statistics [HES]) produced longitudinal data for 7,713 older adults (65 years and older) and 25,864 observations. We divided the sample into three groups by education level: low (no formal qualifications), mid (completed compulsory education), and high (at least some higher education). Four outcomes were examined: annual outpatient appointments, elective inpatient admissions, emergency inpatient admissions, and emergency department (ED) visits. We estimated regressions for the periods 2004‐2005 to 2008‐2009 and 2010‐2011 to 2014‐2015 to examine whether potential education‐related inequalities in hospital use increased after the growth rate for NHS funding slowed in 2010.

**Findings:**

For the study period, our sample of ELSA respondents in the low‐education group made 2.44 annual outpatient visits. In comparison, after controlling for health status, we found that participants in the high‐education group made an additional 0.29 outpatient visits annually (95% confidence interval [CI], 0.11‐0.47). Additional outpatient health care use in the high‐education group was driven by follow‐up and routine appointments. This inequality widened after 2010. Between 2010 and 2015, individuals in the high‐education group made 0.48 (95% CI, 0.21‐0.74) more annual outpatient visits than those in the low‐education (16.9% [7.5% to 26.2%] of annual average 2.82 visits). In contrast, after 2010, the high‐education group made 0.04 (95% CI, −0.075 to 0.001) fewer annual ED visits than the low‐education group, which had a mean of 0.30 annual ED visits. No significant differences by education level were found for elective or emergency inpatient admissions in either period.

**Conclusions:**

After controlling for demographics and health status, there was no evidence of inequality in elective and emergency inpatient admissions among the education groups in our sample. However, a period of financial budget tightening for the NHS after 2010 was associated with the emergence of education gradients in other forms of hospital care, with respondents in the high‐education group using more outpatient care and less ED care than peers in the low‐education group. These estimates point to rising inequalities in the use of hospital care that, if not reversed, could exacerbate existing health inequalities in England. Although the US and UK settings differ in many ways, our results also suggest that a universal health care system would likely reduce inequality in US health care use.

Us policymakers and researchers have often looked to health systems in other countries for inspiration on how to provide care more equitably, efficiently, and effectively.^1^, ^2^, ^3^, ^4^ While there has been a fierce debate about whether and how to expand US health care coverage for many years,^5^, ^6^, ^7^, ^8^, ^9^ recent events have again moved health care toward the top of the US political agenda. The COVID‐19 pandemic has brought into sharp focus those communities unable to access medical care,^10^ and several candidates aspiring to be the Democratic Party's presidential nominee in 2020 advocated for a single‐payer universal health care system (such as “Medicare for All”^11^), a model that is commonplace in other countries. Debates about the merits of a shift toward such a model will likely feature heavily in US politics for years to come.

The UK National Health Service (NHS) is one prominent example of a single‐payer universal health care system. Regular reports from the Commonwealth Fund have found that the United States is regularly at or close to the bottom of international rankings of health care access and equity, whereas the United Kingdom is often at or near the top.^12^ The NHS, however, is not without its own problems: funding growth has been sharply reduced in recent years, and a number of quality indicators reveal increasing signs of strain.^13^ Understanding the extent to which health care inequalities are present within the UK system—and how these have changed over time as funds for growth have been reduced—may provide important lessons for the United States as it seeks to expand health care coverage.

Ensuring that access to health care is based on clinical need, not age, location, or ability to pay, is a founding principle of the English NHS.^14^ This principle was restated in the 2012 Health and Social Care Act, which enshrined in legislation policy objectives to reduce inequalities in both access to care and health care outcomes.^15^ Health inequalities across socioeconomic groups have long been recognized in England (and in other countries, including the United States), with lower socioeconomic status associated with worse health.^16^, ^17^, ^18^, ^19^, ^20^, ^21^ However, the extent of health care inequalities in England is less well understood. This is in large part due to a lack of representative data linking individual characteristics with administrative health care records. Previous research was restricted to investigations of single treatments or specialties,^22^, ^23^, ^24^ studies focused on area‐based characteristics as proxies for individual characteristics,^25^, ^26^ and studies using limited individual health data.^27^

We built upon this work using a new data set linking individual educational attainment, as captured by the English Longitudinal Study of Ageing (ELSA), with administrative hospital records in the Hospital Episode Statistics (HES) data set. We used education as proxy for adult socioeconomic status because it is time invariant in later life and therefore is not determined by health status in older age.^28^ In addition, education itself may have effects on individuals’ behavior and ability to access and interpret health care–related information, as well as doctor‐patient communication.^29^ Using these data, we investigated inequality in the use of four types of hospital care—outpatient, elective inpatient, emergency inpatient, and emergency department (ED)—across three groups defined by education level, before and after controlling for differences in health status across groups. Variation in health care use across groups with similar clinical needs may in part reflect socioeconomic inequalities in access, and it is likely to exacerbate health inequalities across socioeconomic groups. We also examined whether these potential inequalities increased after 2010, when NHS funding increases slowed sharply: the average real annual growth rate of UK health spending was 5.1% between 2003‐2004 and 2009‐2010 and then slowed to 1.1% between 2009‐2010 and 2014‐2015 (Figure 1).^30^

**Figure 1:**
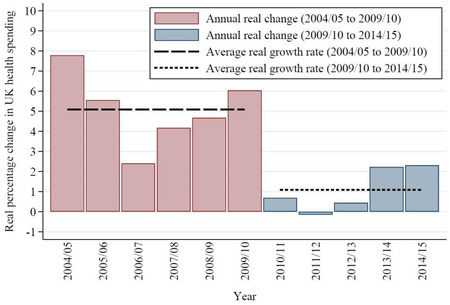
Real Changes in UK Health Spending, 2004‐2005 to 2014‐2015 [Color figure can be viewed at wileyonlinelibrary.com] Figures denote the percentage change in real‐term spending relative to the previous financial year. Source: Authors’ calculations using data from reference 30.

## Background on the NHS and Hospital Use in England

The English NHS is a universal single‐payer health care system. All residents can access care free at the point of use. Copayments are extremely rare and are limited to some means‐ and needs‐tested fixed fees for prescriptions and some aspects of dentistry and optometry. Access to care should therefore be independent of one's ability to pay or any other socioeconomic characteristics.

This article focuses on hospital care. In England, hospital care is provided in the vast majority of cases by large public hospitals staffed by public employees. Patients can access emergency care at any public hospital by visiting an Accident and Emergency department. Patients may then be admitted for inpatient treatment if required.

Preplanned outpatient and inpatient treatment is also provided by the same public hospitals. Patients attend outpatient consultations or treatment at hospitals following a referral from either their primary care doctor/general practitioner (GP) or a hospital consultant. Referrals by a hospital consultant are made either following an ED visit or as follow‐up to a previous planned care encounter. Patients who require further care may then be admitted for a pre‐planned procedure as an inpatient. Access to planned care is regulated by GPs (with referrals limited only to patients meeting certain clinical thresholds) and by waiting times.

Because NHS patients do not pay out of pocket, access to health care should be equal across socioeconomic groups. However, use (and access) may still vary across education groups. In particular, the following differences seem likely to affect health care use/access.

First, informational and cultural barriers may introduce inequalities in health care use. For example, physicians may interact with certain groups differently, which could lead to differences in referral and treatment patterns.^29^, ^31^, ^32^, ^33^ This could occur because physicians are more willing to listen to the complaints of particular groups (and more dismissive of other groups’ concerns), or because members of certain groups find it easier to engage with medical professionals about their conditions and treatment. More generally, better educated patients may find it easier to navigate the NHS system than individuals with less education, and may therefore be more likely to seek an initial referral and attend subsequent appointments.

Second, tastes and preferences for hospital care may vary across socioeconomic groups.^34^ Differences in hospital use may therefore simply reflect differences in the demand for care (even for the same levels of objective health needs), rather than systematic differences in access to care. In this case, variation in hospital use associated with education level should not be interpreted as evidence of unequal access. However, policymakers may still be concerned if different socioeconomic groups have very different expectations of good health.

In addition to the public market, there is a small private market for hospital care in England, which provides some routine, preplanned care to patients who either fund this treatment themselves or have private medical insurance (purchased by the individual or provided by an employer). There is no private market in England for emergency care. Information on privately financed care is extremely scarce. We therefore focused only on use of public hospitals in our study. To the extent that more educated people, who on average have higher incomes, are more likely to use private care, our findings likely underestimated total hospital use among this group. To mitigate this concern, we restricted our sample to adults 65 years and older. Rates of private insurance are much lower in this age group than in the working‐age population. In our sample, 17.5% of the 50‐ to 64‐year‐old population had private health insurance, compared to just 9.8% among those 65 years and older, with rates falling sharply at older ages.

## Methods

### Data Sources

We examined linked data on individuals from the English Longitudinal Study of Ageing (ELSA) and Hospital Episode Statistics (HES). ELSA is a representative panel study of adults in England aged 50 years and above. The study began in 2002‐2003 with 12,099 participants recruited from the 1998‐2001 Health Survey for England. Respondents are interviewed every two years, with the sample refreshed periodically to maintain representativeness of the wider population. The study collects detailed self‐reported information on socioeconomic characteristics (including measures of income, wealth, and education) and the health and well‐being of respondents. During the first seven waves of the study (2002‐2003 to 2014‐2015), 18,529 people participated, appearing in 72,933 observations.

Although ELSA includes detailed measures of health, the study does not capture information about participants’ hospital use. We therefore combined the survey responses with administrative hospital records contained in HES for respondents who consented to these records being linked. NHS Digital (the organization that manages NHS data) used National Insurance numbers (a unique number allocated to every adult ever in the labor market) to link the records and then shared anonymized records with researchers. Eighty percent of participants (14,789 of 18,529), accounting for 85% of observations (62,046 of 72,933), gave consent for their records to be linked. We dropped nonconsenting participants from our sample.

ELSA data include cross‐sectional inverse probability weights that make it possible to scale observations to generate a nationally representative level. Consenting and nonconsenting participants were broadly similar but differed along some dimensions, including education level, gender, and certain health characteristics. We therefore reweighted the observations to allow selection into the consenting sample on the basis of observable characteristics. We used a probit regression to estimate the relationship between consent and the full set of controls used in our baseline statistical analysis and used these coefficients to predict participation in the sample. We multiplied predicted participation by the inverse of the entire‐sample weights to produce updated inverse probability weights in the consenting sample.

HES contains information on all NHS‐funded outpatient visits (between 2003‐2004 and 2016‐2017), inpatient admissions (between 1997‐1998 and 2016‐2017) and ED visits (between 2007‐2008 and 2016‐2017). The records include the dates of visits and admissions. We used these dates to create variables that counted the number of outpatient appointments, elective and emergency inpatient admissions, and ED visits that took place for a participant in the year prior to each ELSA interview. Elective and emergency inpatient admissions were defined by the admission method recorded in HES. Elective admissions were defined as admissions when the date of the decision to admit differed from actual admission date, whereas emergency admissions were not planned.

For most of our analysis, we used data from ELSA waves two through seven (2004‐2005 to 2014‐2015) in our final sample. HES data are unavailable for ED visits prior to 2007‐2008, so our sample was limited to ELSA waves four through seven (2008‐2009 to 2014‐2015, henceforth referred to as the “ED sample”).

In our main analysis, we excluded respondents under the age of 65 (20,442 observations and 6,800 people from the full sample; 13,055 observations and 5,175 people from the ED sample). We also excluded respondents with missing variables (386 observations or 1.5% of those 65 years and older).

We focused on the population 65 years and older for a number of reasons. First, limiting our analysis to this group allowed us to focus on the most common users of health care and reduced the proportion of individuals who had no hospital visits of any type in the year prior to their ELSA interview: 56% of participants between the ages of 50 and 64 years had no hospital use in the year prior to interview, compared to 40% of those 65 years and older.

Second, in the population we selected, most participants were retired—for most of the study period, with a majority retiring at age 65 years for men and 60 years for women. Work provides an important channel through which inequalities in health care use may be generated: working‐age individuals with low levels of education may be more likely to have less‐flexible hours or less job security than individuals with higher levels of education, and may therefore find it more difficult to attend doctor appointments. We chose to examine inequalities in use among participants 65 years and older to shut down this mechanism.

Finally, as noted previously, private medical insurance rates are significantly higher in the working‐age population compared to the older group. By focusing on the older population, we reduced concerns that widespread use of (unobserved) private medical care would cause variation across the education groups.

**Figure 2:**
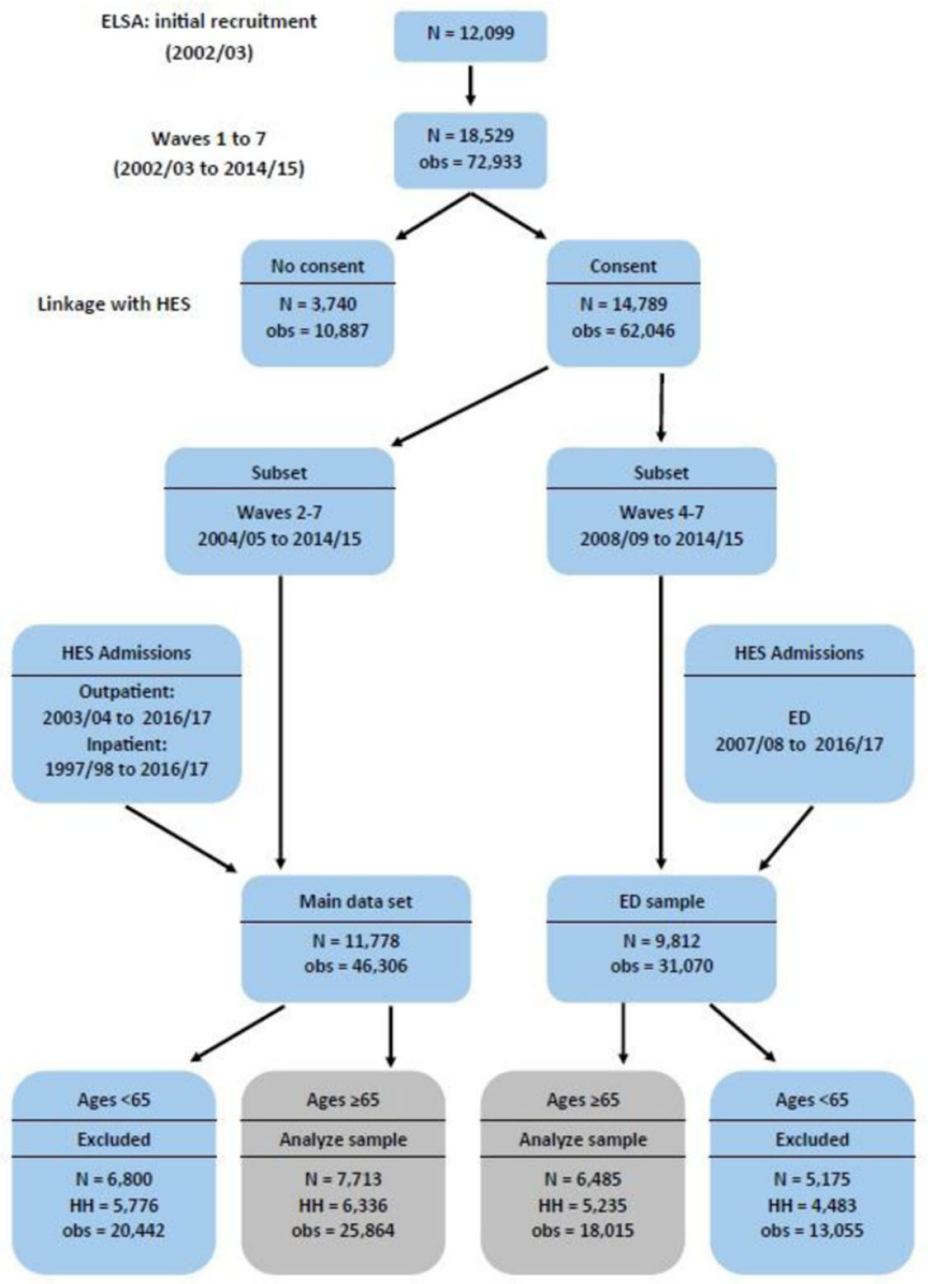
Data Structure [Color figure can be viewed at wileyonlinelibrary.com] Abbreviations: ED, emergency department; ELSA, English Longitudinal Study of Ageing; HES, Hospital Episode Statistics; HH, household; obs, observations.

Our final sample included 25,864 observations of 7,713 individuals (in 6,336 households) who were interviewed between June 2004 and May 2015. The ED sample consisted of 18,015 observations of 6,485 individuals (in 5,235 households) who were interviewed between June 2008 and May 2015. Figure 2 summarizes the data structure and sample construction.

ELSA records individuals’ level of education, which we transformed into a three‐point scale to create the groups in our sample:

- Low: No formal qualifications
- Mid: NVQ1/NVQ2/NVQ3, O level, A level or equivalent (comparable with 10th to 12th grade education in the United States)
- High: NVQ4/NVQ5, higher education below degree, or degree equivalent (comparable with college degrees or similar vocational qualifications in the United States)

### Statistical Analysis

#### Educational Inequalities in Hospital Use Between 2004‐2005 and 2014‐2015

We used multivariable regression to examine the relationship between the number of annual hospital visits of each type and education group after controlling for a range of demographic and observed health measures that are recorded in the ELSA survey. We included survey wave indicator variables in all regressions to control for common national trends in use of care over the period. We weighted all regressions using the weights described previously. We used robust standard errors clustered at the household level in all regressions. Stata version 14 was used for all analysis.

Demographic control variables included participant age, sex, ethnicity, interactions between these variables, and indicators of living as a couple and being engaged in paid work. Health variables included:

- Self‐reported health on a three‐point scale (excellent/very good, good, and fair/poor).
- Number of mobility difficulties, number of difficulties with activities of daily living (ADLs), and number of difficulties with instrumental activities of daily living (IADLs), all of which were additionally interacted with sex.
- Indicator variables capturing whether the respondent had a long‐standing illness. In cases of where the long‐standing illness was limiting, indicator variables further captured whether the participant received formal or informal social (long‐term) care and whether they died in the two years after the ELSA interview.
- An indicator variable capturing whether the participant reported a score of four or more on the eight‐point Centre for Epidemiologic Studies Depression Scale (CES‐D).^35^, ^36^
- Indicator variables recording whether the respondent reported ever having been diagnosed with 19 separate health conditions. These indicators comprise the full range of specific health conditions collected in ELSA: lung disease, asthma, arthritis, osteoporosis, cancer, Parkinson's disease, psychiatric problems, Alzheimer's disease, dementia, blood disorders, hypertension, angina, heart attack, congestive heart failure, heart murmur, heart arrhythmia, diabetes, stroke, and high cholesterol.

Our set of control variables included an array of self‐reported health measures, intended to capture as many differences as possible in observed health status, and therefore the need for health care, across the education groups. However, a number of these health measures are highly subjective, and the relationship between subjective and objective health measures may vary across education groups due to differences in perceptions, preferences, and available resources to mitigate the impact of health conditions. These include self‐reported general health (rated as excellent/very good, good, or fair/poor), a self‐reported score on the CES‐D depression scale, and whether an individual self‐reports a longstanding illness, or a longstanding and limiting illness. This is more likely to be the case for broader questions about general health than for specific questions about whether an individual has been diagnosed with a particular condition.

In particular, perceptions of general health may be very different across education groups. For example, individuals with low levels of education might be more likely than those with high levels to accept certain health conditions as normal. If that is true, the individuals with objectively poor health status in the low‐education group might rate their health more positively than their more educated peers would. In contrast, people in the high‐education group might have more resources than those in the low‐education group to mitigate the negative effects of such health conditions, and therefore report their health at a higher level even if they have specific health conditions.

These differences in perception could therefore capture non‐health differences across education groups. We aimed to examine whether and how hospital use varied across education groups among individuals with the same objective health status, and controlling for these subjective measures of health status may in fact account for some of the variation in use that we wished to study. Similarly, self‐reported data on receiving social (long‐term) care could have reflected differences in access to, or preferences regarding, home‐based help, rather than measuring underlying medical need. We therefore repeated the analysis excluding the subjective health and self‐reported social (long‐term) care receipt variables to examine whether our results changed when these variables were omitted.

To further examine inequalities in outpatient care, we also repeated the multivariable analysis separately by whether the visit was for a first or follow‐up appointment and by referral source (GP, hospital consultant, or other); and by priority type (routine, urgent, two‐week cancer referral, or other).

In our baseline analysis, health care use was modeled as a linear outcome. This provided easily interpretable coefficients and absolute differences in utilization among groups. However, the number of hospital visits was limited to non‐negative values, with a large number of zeros (respondents with no hospital use), and very few large numbers. We therefore repeated our main analysis using a zero‐inflated negative binomial regression model as a robustness test, using the same set of control variables as in the linear model for both the first and second stage.

##### Trends in Educational Inequalities Before and After NHS Funding Growth Slowed in 2010

We estimated multivariable regressions for each care type separately for two distinct periods (2004‐2005 to 2008‐2009 vs 2010‐2011 to 2014‐2015) to examine whether any inequalities in hospital use across education groups changed after 2010, when NHS funding growth slowed. We controlled for the same demographic and health measures as in the pooled analysis. All regressions were weighted, and standard errors clustered at the household level.

## Findings

### Educational Inequalities in Hospital Use Between 2004‐2005 and 2014‐2015

Table 1 shows the characteristics of ELSA respondents by education group. There were no obvious differences in outpatient or inpatient emergency inpatient care across education groups. There was a small, negative education gradient in elective inpatient care and ED visits. These patterns held when we adjusted for age, sex, ethnicity, marital status, and employment status (see Figure A1 and Table A2 in the Appendix).

**Table 1:** Hospital Use, Demographic Characteristics, and Self‐Reported Health, by Level of Education, 2004‐2015^a^

	Education Level				
	Low	Mid	High	All	
	Mean	Mean	Mean	Mean	SD
**Hospital use** *No. in previous year*:					
Outpatient visits	2.44	2.48	2.43	2.45	4.25
Emergency inpatient admissions	0.38	0.35	0.35	0.36	1.49
Elective inpatient admissions	0.20	0.14	0.12	0.16	0.55
ED visits^b^	0.30	0.24	0.21	0.25	0.74
**Demographics**					
Age, y^c^	75.6	73.6	73.1	74.2	6.88
Female, %	0.63	0.55	0.42	0.55	0.50
Nonwhite, %	0.02	0.02	0.02	0.02	0.14
In a couple, %	0.52	0.64	0.71	0.61	0.49
In paid work, %	0.05	0.10	0.12	0.09	0.29
**Health**					
*Percentage reporting*:					
Very good health	0.23	0.34	0.42	0.32	0.47
Good health	0.31	0.35	0.36	0.34	0.47
Poor health	0.46	0.31	0.22	0.34	0.47
Long‐standing illness	0.66	0.60	0.58	0.62	0.49
Limiting and long‐standing illness	0.48	0.39	0.35	0.41	0.49
Formal long‐term care receipt	0.08	0.06	0.05	0.07	0.25
Informal long‐term care receipt	0.29	0.20	0.13	0.21	0.41
Score ≥4 on CES‐D	0.18	0.13	0.09	0.14	0.35
Difficulties with mobility	3.09	2.29	1.75	2.45	2.69
Difficulties with ADLs	0.62	0.43	0.32	0.48	1.05
Difficulties with IADLs	0.78	0.46	0.34	0.55	1.19
Proportion of respondents who die within 2 years of interview	0.07	0.05	0.04	0.06	0.23
No. of observations	9,611	9,688	6,565	25,864	
No. of observations (ED sample)^b^	6,088	7,025	4,902	18,015	

Abbreviations: ADL, activity of daily living; CES‐D, Centre for Epidemiologic Studies Depression Scale; ED, emergency department; IADL, instrumental activity of daily living.

^a^ Mean values are shown for each education group. CES‐D is an 8‐point scale to assess depression. Education is classified as low (no formal qualifications), mid (completed compulsory education), or high (at least some higher education). Summary statistics for self‐reported specific diagnoses are included in Table 1A of the Appendix.

^b^ ED data are available only for wave 4 (2008‐2009) onward.

^c^ The sample is restricted to participants 65 years and older.

There was a strong, positive correlation between education level and various measures of health. Compared with participants in the mid‐ and high‐education groups, those in the low‐education group were more likely to be in poor health (and less likely to be in very good or good health), more likely to report a long‐standing and limiting illness, and more likely to report four or more depressive symptoms on the CES‐D; those in the low‐education group also had higher average numbers of mobility difficulties, ADLs, and IADLs than other respondents.

**Figure 3:**
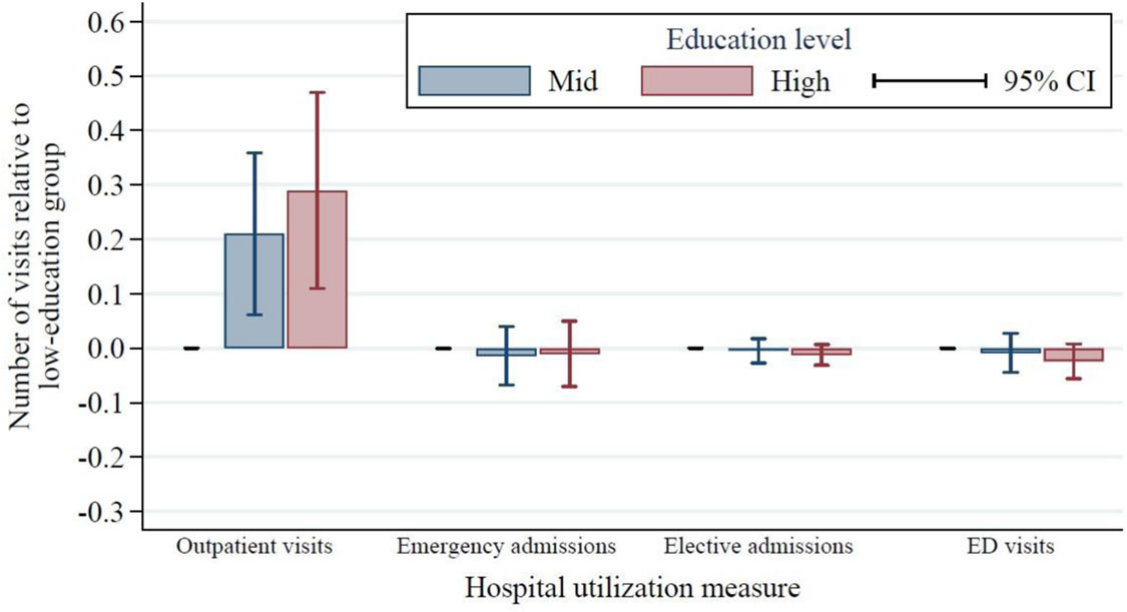
Hospital Use Between 2004‐2005 and 2014‐2015 by Education Group, Adjusted for Demographic and Health Status^a^ [Color figure can be viewed at wileyonlinelibrary.com] ^a^Education is classified as low (no formal qualifications), mid (completed compulsory education), or high (at least some higher education). All hospital use measures were calculated relative to use among those in the low‐education group, adjusting for English Longitudinal Study of Ageing wave (year), individual demographic characteristics (age, age^2^, sex, being nonwhite, being in a couple, being in paid work, and interaction effects between sex and age and sex and being nonwhite), and individual health characteristics (self‐reported general health [fair/poor, good, or very good], scoring ≥4 on the 8‐item Centre for Epidemiologic Studies Depression Scale, difficulties with mobility, difficulties with activities of daily living, difficulties with instrumental activities of daily living, reporting a long‐standing illness, reporting a long‐standing and limiting illness, being in receipt of informal long‐term care, being in receipt of formal long‐term care, whether the individual died in the two years following the interview, and whether the individual was ever diagnosed with lung disease, asthma, arthritis, osteoporosis, cancer, Parkinson's disease, psychiatric problems, Alzheimer's disease, dementia, a blood disorder, hypertension, angina, heart attack, congestive heart failure, heart murmur, heart arrhythmia, diabetes, stroke, or high cholesterol). Data on emergency department (ED) visits were available only for 2008‐2009 onward.

Figure 3 and Table 2 show that, after controlling for these differences in mental and physicial health, there was no statistically significant relationship between education level and inpatient treatment (emergency or elective) or ED visits. The direction and magnitude of the coefficients in each case indicated a modest negative education gradient in use of these services, but the estimates were rather imprecise, with relatively wide confidence intervals (CIs). For instance, our point estimates suggested that respondents in the high‐education group made 8.0% fewer ED visits than the low‐ education group, but the confidence interval spanned from –18.7% to 2.7% (see Table 2). A similar pattern was found for emergency and elective inpatient care admissions. Although these results were statistically imprecise, they did point, at the very least, toward the absence of any notable positive education gradient in the use of these types of hospital care.

**Table 2:** Estimated Relationships Between Education Level and Use of Hospital Services, Adjusted for Demographic and Health Characteristics, 2004‐2005 to 2014‐2015^a^

	No. of Hospital Visits in the Last Year (95% CI)			
	**Outpatient**	**Emergency Inpatient**	**Elective Inpatient**	**Emergency Department**
Panel A: Relative to low‐education group				
Education level:				
Low	0.0 (Ref)	0.0 (Ref)	0.0 (Ref)	0.0 (Ref)
Mid	0.210 (0.061 to 0.359)	−0.014 (−0.067 to 0.039)	−0.005 (−0.027 to 0.018)	−0.009 (−0.044 to 0.027)
High	0.290 (0.110 to 0.470)	−0.011 (−0.071 to 0.049)	−0.012 (−0.032 to 0.007)	−0.024 (−0.056 to 0.008)
Panel B: As a proportion of use in low‐education group				
Mean no. of visits in low‐education group	2.44	0.38	0.20	0.30
Education level:				
Mid	8.6% (2.5% to 14.7%)	−3.7% (−17.6% to 10.3%)	−2.5% (−13.5% to 9.0%)	−3.0% (−14.7% to 9.0%)
High	11.9% (4.5% to 19.3%)	−2.9% (−18.7% to 12.9%)	−6.0% (−16.0% to 3.5%)	−8.0% (–18.7% to 2.7%)

^a^ Education is classified as low (no formal qualifications), mid (completed compulsory education), or high (at least some higher education). Data in Panel A show the number of hospital visits relative to those with low education in the year prior to the ELSA interview. Data in Panel B show the mean number of hospital visits of each type in the year prior to the interview for those in the low‐education group, and the coefficients from Panel A as a proportion of that sample mean. All data were calculated using the full model, adjusting for wave (year), individual demographic characteristics (wave [year] and individual demographic and health characteristics: age, age^2^, sex, being non‐white, being in a couple, being in paid work, and interaction effects between sex and age and sex and being nonwhite), and individual health characteristics (self‐reported general health [fair/poor, good, or very good], scoring ≥4 on the 8‐item Centre for Epidemiologic Studies Depression scale, difficulties with mobility, difficulties with activities of daily living, difficulties with instrumental activities of daily living, reporting a long‐standing illness, reporting a long‐standing and limiting illness, being in receipt of informal long‐term care, being in receipt of formal long‐term care, whether the individual died in the two years following the interview, and whether the individual had ever been diagnosed with lung disease, asthma, arthritis, osteoporosis, cancer, Parkinson's disease, psychiatric problems, Alzheimer's disease, dementia, a blood disorder, hypertension, angina, heart attack, congestive heart failure, heart murmur, heart arrhythmia, diabetes, stroke, or high cholesterol). The 95% confidence intervals (CIs) were calculated using robust standard errors clustered at the household level.

In contrast, there was a large positive education gradient in the number of outpatient visits. Compared to respondents in the low‐education group, those in the high‐education group made 0.29 (95% CI, 0.11‐0.47) more outpatient visits annually, and those in the mid‐education group made 0.21 (95% CI, 0.06‐0.36) additional outpatient visits each year (Table 2). The mean number of annual outpatient attendances in the low‐education group was 2.44; therefore, the mean differences in annual outpatient visits were 11.9% (95% CI, 4.5%‐19.3%) and 8.6% (95% CI, 2.5%‐14.7%) for the high‐ and mid‐ education groups, respectively. Again, the confidence intervalswere relatively wide, but the point estimates indicated policy relevant differences in the use of outpatient care across education groups.

Table 3 shows that the low‐education group had the lowest numbers of first encounters and follow‐up outpatient visits, after adjusting for demographic and health characteristics. In proportional terms, the magnitude of the education gradient was similar in each case. In absolute terms, however, the difference in use of outpatient care across education groups was driven by follow‐up care: Compared to the low‐education group, the high‐education group had 0.08 (95% CI, 0.04‐0.12) more first encounters per year and 0.21 (95% CI, 0.05‐0.37) additional follow‐up visits annually.

**Table 3:** Estimated Relationships Between Education and Use of Outpatient Hospital Services for First Encounters and Follow‐Up Care, Adjusted for Demographic and Health Characteristics, 2004‐2005 to 2014‐2015^a^

	No. of First Outpatient Encounters in the Last Year (95% CI)				
	**By Referral Source**				
	**All**	**GP**	**Hospital**	**Other**	**Follow‐Up Visits**
Panel A: Relative to low‐education group					
Education level:					
Low	0.0 (Ref)	0.0 (Ref)	0.0 (Ref)	0.0 (Ref)	0.0 (Ref)
Mid	0.057 (0.021 to 0.093)	0.030 (0.008 to 0.052)	0.023 (0.004 to 0.042)	0.004 (−0.009 to 0.016)	0.153 (0.024 to 0.283)
High	0.080 (0.040 to 0.121)	0.046 (0.021 to 0.070)	0.027 (0.006 to 0.049)	0.007 (−0.007 to 0.021)	0.210 (0.051 to 0.370)
Panel B: As a proportion of use in low‐education group					
Mean no. of visits in low‐education group	0.64	0.35	0.20	0.08	1.80
Education level:					
Mid	8.9% (3.3% to 14.5%)	8.6% (2.3% to 14.9%)	11.5% (2.0% to 21.0%)	5.0% (−11.3% to 20%)	8.5% (1.3% to 15.7%)
High	12.5% (6.3% to 18.9%)	13.1% (6.0% to 20.0%)	13.5% (3.0% to 24.5%)	8.8% (–8.8% to 26.3%)	11.7% (2.8% to 20.6%)

^a^ Education is classified as low (no formal qualifications), mid (completed compulsory education), or high (at least some higher education). Data in Panel A show the number of hospital visits relative to those in the low‐education group in the year prior to the English Longitudinal Study of Ageing interview. Data in Panel B show the mean number of hospital visits of each type in the year prior to the interview for individuals in the low‐education group and the coefficients from Panel A as a proportion of that sample mean. All data were calculated using the full model, adjusting for wave (year), individual demographic characteristics (age, age^2^, sex, being nonwhite, being in a couple, being in paid work, and interaction effects between sex and age and sex and being nonwhite), and individual health characteristics (self‐reported general health [fair/poor, good, or very good], scoring ≥4 on the 8‐item Centre for Epidemiologic Studies Depression Scale, difficulties with mobility, difficulties with activities of daily living, difficulties with instrumental activities of daily living, reporting a long‐standing illness, reporting a long‐standing and limiting illness, being in receipt of informal long‐term care, being in receipt of formal long‐term care, whether the individual died in the 2 years following the interview, and whether the individual was ever diagnosed with lung disease, asthma, arthritis, osteoporosis, cancer, Parkinson's disease, psychiatric problems, Alzheimer's disease, dementia, a blood disorder, hypertension, angina, heart attack, congestive heart failure, heart murmur, heart arrhythmia, diabetes, stroke, or high cholesterol).

The differences in outpatient visits are mostly explained by differences in routine outpatient appointments rather than more urgent care (see Table A3 in the Appendix). The high‐education group made 0.25 (95% CI, 0.08‐0.42) more routine visits annually than the low‐education group. There were no statistically significant differences in urgent, two‐week (cancer), or unknown priority referrals between the two groups. This indicates that educational inequalities in the use of outpatient hospital care were driven by the groups’ differential use of routine follow‐up care. For more urgent appointments, such as “two‐week” referrals for cancer symptoms, our estimated coefficients for the mid‐ and high‐education groups were close to zero with relatively narrow confidence intervals.

### Robustness Checks

Our main analyses were based on hospital use in the year prior to survey interview in order to avoid concerns around competing risks and to make our analyses comparable to previous work using data on self‐reported health care use (which, by necessity, was based on past patterns of use). One concern with this approach is that self‐reported health status in ELSA, which we used as a control, could be endogenous to the amount of health care received over the past year. We therefore tested the robustness of our findings with a measure of hospital use in the year *after* the interview. As shown in Table A5 of the Appendix, this test left our key results broadly unchanged: Respondents in the high‐education group made an additional 0.25 (95% CI, 0.06‐0.44) outpatient visits annually relative to those in the low‐education group; this compares to 0.29 (95% CI, 0.11‐0.47) in our baseline analysis (Table 2). The robustness test analysis, like the baseline analysis, found no education gradient in the use of emergency or elective inpatient care. The coefficient on ED visits remained negative but doubled in magnitude compared to our baseline analysis, with the high‐education group making 0.05 (95% CI, 0.01‐0.09) fewer annual visits than the low‐education group. Note that by examining use in the year after the ELSA interview, rather than in the year before, we were, by definition, analyzing patterns of hospital use in a later period, which we would expect to affect our results if the education gradient changed over time—an issue we discuss in the following section.

Our findings were also robust when analyzed using alternative modeling choices. For each of the four types of care, our results were essentially unchanged when we used a zero‐inflated negative binomial (ZINB) regression model. In our baseline analysis, when the dependent variable was modeled as a linear outcome, individuals in the high‐education group made 0.29 additional outpatient visits annually compared to those in the low‐education group, equivalent to 11.9% of the sample mean among that group (2.44). Using the ZINB model, the incidence rate ratio was 1.103 (see Table A6 in the Appendix), indicating that the high‐education group made 10.3% more outpatient visits than the low education group. This finding was qualitatively similar to, and comfortably within the confidence interval of, our main results. A similar pattern was found for our other outcomes, so the policy conclusions from our analysis were unchanged.

As noted previously, some of the control variables included in the full model might not only reflect differences in the need for care across education groups but also capture other differences across groups (such as self‐perception of health status). We therefore analyzed how the estimates for our main results (on outpatient care) changed when we excluded the self‐reported health measures most likely to be subject to differential reporting. When these control variables were excluded, the magnitude of the coefficients was reduced somewhat (from 0.29 to 0.17 additional outpatient visits annually for the high‐education group relative to the low‐education group). However, there remained a positive education gradient in the use of outpatient care that was significant at the 10% level (see Table A2 of the Appendix).

### Trends in Educational Inequalities Before and After NHS Funding Growth Slowed in 2010

**Figure 4:**
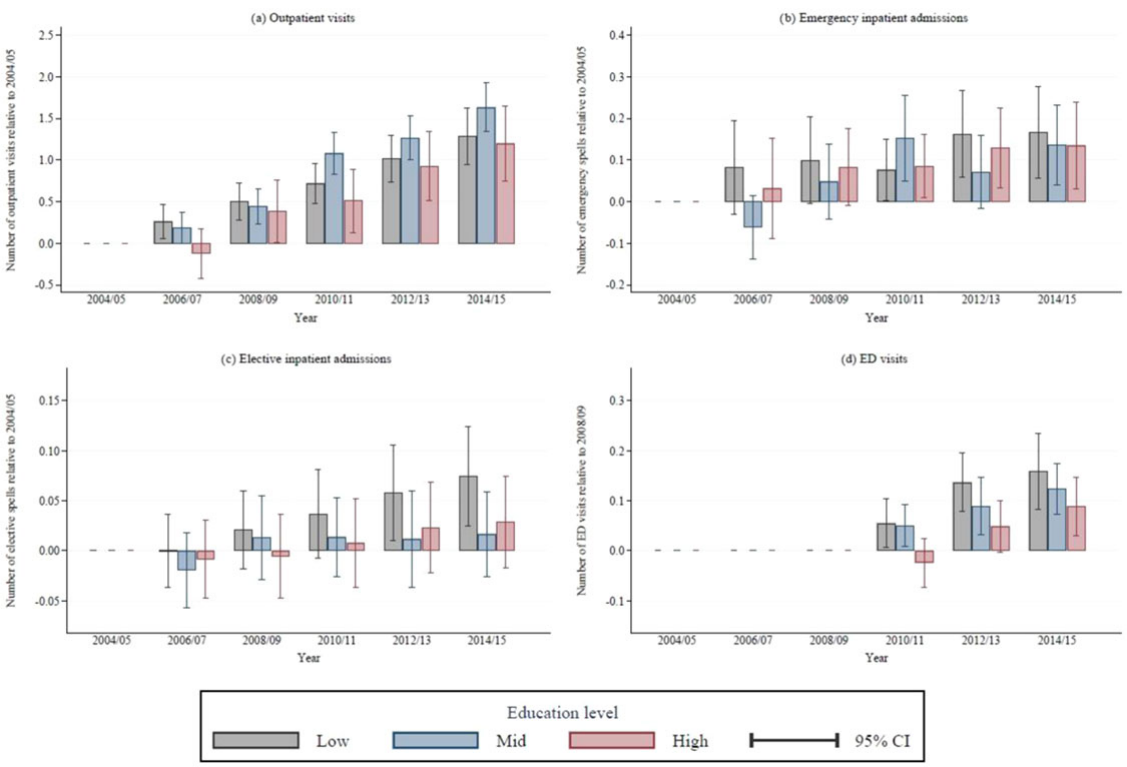
Trends in Use of Hospital Care Between 2004‐2005 and 2014‐2015 by Care Type and Education Level, Adjusted for Demographic Characteristics^a^ [Color figure can be viewed at wileyonlinelibrary.com] ^a^Education is classified as low (no formal qualifications), mid (completed compulsory education), or high (at least some higher education). All hospital use measures were calculated relative to use in English Longitudinal Study of Ageing wave 2 (2004‐2005), after adjusting for individual demographic characteristics (age, age^2^, sex, being nonwhite, being in a couple, being in paid work, and interaction effects between sex and age and sex and being nonwhite). Emergency department (ED) data were available only from 2008‐2009 onward.

Figure 4 shows the changes in use of each type of hospital care for the education groups over time, after adjusting for age, sex, ethnicity, marital status, and whether the respondent worked. Panels A, B, and C show changes relative to 2004‐2005 for outpatient encounters, emergency inpatient admissions, and elective inpatient admissions, respectively. Panel D shows changes relative to 2008‐2009 for ED visits. Use of each service increased over time for all three education groups. However, there were no significant differences across education groups in the growth of any of the four types of care over the entire period.

**Figure 5:**
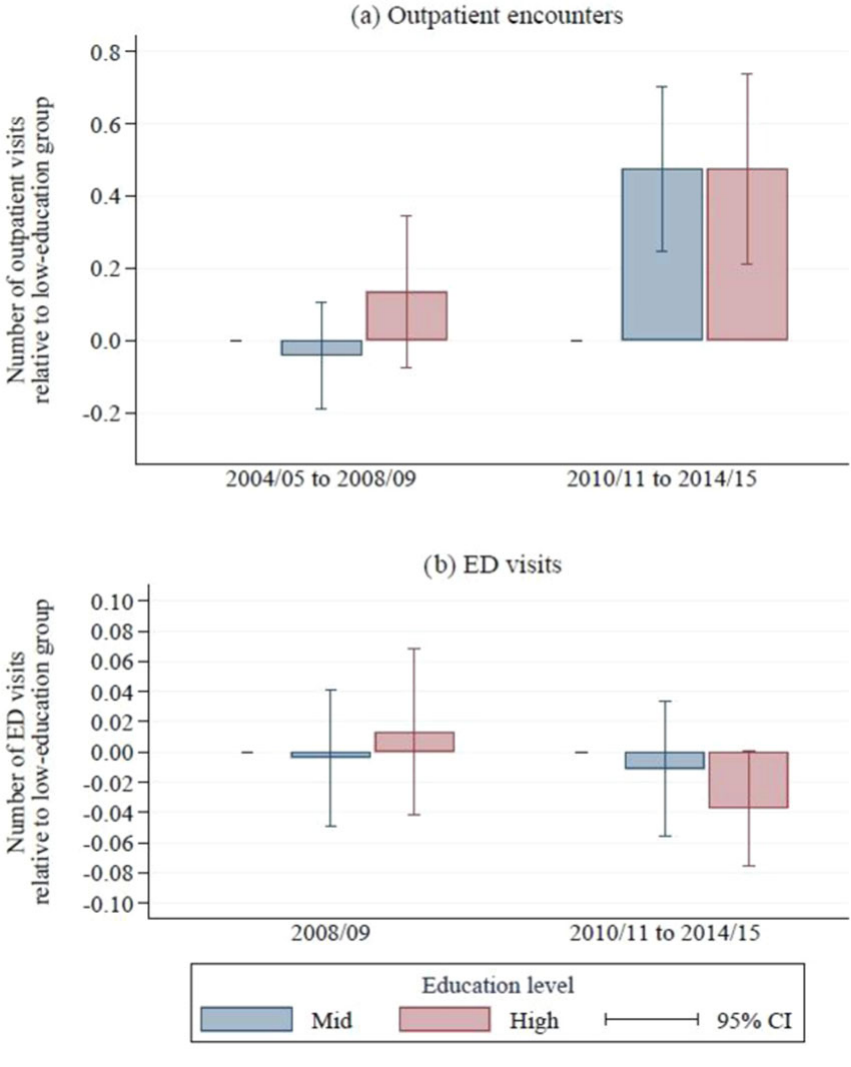
Relative Outpatient Hospital Use, 2004‐2009 vs 2010‐2015, by Education Group, Adjusted for Demographic and Health Characteristics^a^ [Color figure can be viewed at wileyonlinelibrary.com] ^a^Education is classified as low (no formal qualifications), mid (completed compulsory education), or high (at least some higher education). All measures of hospital use are calculated relative to use among those in the low‐education group, adjusting for English Longitudinal Study of Ageing wave (year), individual demographic characteristics (age, age^2^, sex, being non‐white, being in a couple, being in paid work, and interaction effects between sex and age and sex and being nonwhite), and individual health characteristics (self‐reported general health [fair/poor, good, or very good], scoring ≥4 on the 8‐item Centre for Epidemiologic Studies Depression Scale, difficulties with mobility, difficulties with activities of daily living, difficulties with instrumental activities of daily living, reporting a long‐standing illness, reporting a long‐standing and limiting illness, being in receipt of informal long‐term care, being in receipt of formal long‐term care, whether the individual died in the two years following the interview, and whether the individual was ever diagnosed with lung disease, asthma, arthritis, osteoporosis, cancer, Parkinson's disease, psychiatric problems, Alzheimer's disease, dementia, a blood disorder, hypertension, angina, heart attack, congestive heart failure, heart murmur, heart arrhythmia, diabetes, stroke, or high cholesterol).

Figure 5 illustrates how controlling for underlying differences in physical and mental health affected the pattern of education‐associated inequalities over time. Panel A shows that, after adjusting for differences in physical and mental health, there were no significant differences in outpatient use across education groups between 2004‐2005 and 2008‐2009. However, between 2010‐2011 and 2014‐2015, significant differences developed in use of outpatient care between the low education group and the other groups. In this period, the mean number of annual outpatient visits in the low‐education group was 2.82, whereas respondents in the mid‐ and high‐education groups had, respectively, an additional 0.47 (95% CI, 0.25‐0.70) and 0.48 (95% CI, 0.21‐0.74) outpatient encounters annually.

Panel B of Figure 5 shows a different trend for ED visits. In 2008‐2009 (the only year before 2010 for which we have ED data), there were no significant differences in the number of visits across education groups (albeit, with wide confidence intervals). In the 2010–2015 period, there is some indication that a negative education gradient may have emerged. Respondents in the high‐education group had 0.04 (95% CI, −0.001 to 0.075) *fewer* ED visits than those in the low‐education group. This result, which is statistically significant at the 10% level, is equivalent to 11.3% (95% CI, −0.3% to 23.0%) of the mean 0.33 annual ED visits for low education respondents in that period. Though this finding is not conclusive, when combined with the results for ED use in the year *after* the ELSA interview (as described in the previous section and shown in Table A5 of the Appendix), it suggests that in the years after 2010, respondents in the low‐education group used *more* ED care than their peers in the high‐education group.

There were no significant differences in the number of elective or emergency inpatient admissions in either period (see Table A4 of the Appendix).

## Discussion

Overall, this detailed analysis of inequalities in health care use in an established universal health care system indicates that patients with low levels of education are using less outpatient care than their more educated peers, after taking into account differences in their underlying health. However, use of core inpatient services does not vary in this way after controlling for health status differences.

The differences in outpatient use arise largely from differential use of follow‐up and routine care (rather than first encounters and/or urgent referrals). This finding might be explained by differences in preferences for nonurgent health care across education groups, with a stronger preference for this type of care among those with higher levels of education.

Differences in clinical behavior, with GPs and hospital consultants less likely to refer those in lower education groups for routine treatment, could also play an important role. Highly educated individuals could simply find it easier to navigate the complex NHS bureaucracy. If that were true, reforms to the English NHS in the 2000s, which were designed to give patients more choice over where they received treatment, could have exacerbated this difference. For instance, well‐educated patients may have taken more active roles than those with less education in deciding the location of their initial outpatient appointments and therefore selected consultants with shorter waiting times. Subsequently, individuals from the high‐education group might have felt more involved with their care and thus continued to attend (or push for) follow‐up appointments, when their less educated peers did not.

The difference in the use of outpatient and ED across education groups has also grown over time. Prior to 2010, there were no significant differences among the groups in the use of all types of care after controlling for differences in underlying health. After 2010, during a sharp slowdown in the rate of NHS funding increases, participants in the mid‐ and high‐education groups made greater use of outpatient care than their peers in the low‐education group. Although this study could not identify the precise mechanism behind this change, the trend was consistent with expectations that more educated individuals are better able to access services and navigate health systems when hospital services are being rationed, and it is consistent with recent Organisation for Economic Co‐operation and Development (OECD) research findings that income‐related inequalities in health care use have been rising in the United Kingdom.^37^

When we controlled for underlying health status, we also found that after 2010, participants in the low‐education group visited EDs more often than those with higher levels of education. Increasing use of emergency services among those in the low‐education group over time could potentially be explained by their reduced access to GP and other primary care services as funding settlements tightened—when primary care is less available, patients may turn to emergency services instead. Previous work has shown that individuals of lower socioeconomic status are heavier users of primary care,^38^ and such services have been under increasing pressure in recent years.^39^, ^40^, ^41^ The primary care sector has faced problems with recruitment and, especially, retention of physicians and other health care professionals. In the case of physicians, retention challenges have been attributed to excessive workload, increasing demands from the government and regulators, and changes to taxation of pension contributions.^42^, ^43^, ^44^

Taken together, our results suggest that the universal health care system in England does a reasonable job at reducing differences in the use of core health care services across different socioeconomic groups. However, some differences persist, and these have become more evident during a period of financial restraint.

Our finding of no socioeconomic gradient in use of inpatient care is in line with results from previous studies of the UK health care system.^37^, ^38^, ^45^ A similar lack of gradient in probability of (self‐reported) hospitalization is found in many, but not all, developed countries.^37^

In the cases where we found an education gradient in health care use, it is difficult to directly compare the magnitude of our estimates with those in previous research on the United Kingdom or other countries because many studies focus on income‐related inequalities or report only concentration indices or odds ratios, which do not allow for the calculation of absolute differences between groups. Our estimates on outpatient care seem to be broadly consistent with those recently made by the OECD in a survey‐based study, which found that, after adjusting for demographic characteristics and education level, the richest quintile of individuals had around 24% more visits to a specialist in the previous four weeks than those in the poorest quintile (0.52 vs 0.42).^37^ International comparisons typically find that income‐related inequalities in the use of specialist care in the United Kingdom are less pronounced than in other European countries.^46^ Data limitations inhibit like‐for‐like comparisons between the United States and other nations, but the evidence strongly suggests that socioeconomic disparities in access to and use of health care are wider in the United States than elsewhere.^20^, ^47^, ^48^, ^49^

Although our analysis was specific to the English setting, and one should be cautious when extrapolating such findings to other countries’ health care systems, our findings do suggest that one consequence of a move toward universal health care in the United States would be reduced inequalities in use of health care across socioeconomic groups. Universal health care would not, however, be a panacea. The English NHS does a good job of limiting differences in the use of inpatient hospital services, but we found strong evidence that more educated individuals make greater use of outpatient (specialist) care, which could play an important role in exacerbating future health inequalities. Some education‐related differences in health care use identified in our study could be due to factors specific to the English NHS system—such as referral behavior by GPs. However, some of the differences we found could be driven by variations in preferences for health care across socioeconomic groups—and that is likely relevant to the US setting.

Our analysis raises questions for policymakers about the extent to which measures to reduce inequalities in *access* will also reduce inequalities in health care *use* and *outcomes*. A shift to a universal health care system in the United States might reduce differences in the use of health care across socioeconomic groups, but it seems unlikely to eliminate all health disparities. Our findings suggest that this concern is particularly pertinent when funding constraints are tighter, pointing to the need to ensure that appropriate funding is made available for such a system.

### Strengths and Limitations

To our knowledge, this is the first study to combine administrative data on hospital use with detailed, individual data on the health and socioeconomic characteristics of patients to study patterns of use of hospital care among a nationally representative sample in England. This provides a large advance in data quality and scope relative to previous work.

Our findings are consistent with previously documented research on socioeconomic inequalities in hospital use across a range of conditions. Relative to this previous work, our work has two particularly notable strengths. First, previous studies have often focused on inequalities in the use of single treatments of specialties.^22^, ^23^, ^24^ Restricting attention to narrower clinical areas of medicine is important for identifying variation in those specific areas, but such studies are likely to miss wider trends in the use of NHS hospitals. This study used data covering the entire NHS hospital system over a 10‐year period and therefore captured a broader scope of treatment.

Second, previous work examining differences in health care use associated with socioeconomic characteristics often relied on area‐based characteristics to serve as proxies for individual attributes.^25^, ^26^ When socioeconomic or health characteristics are averaged over geographical areas (even if those areas are small), one risks misclassifying patient attributes and removes important variation from the study. In contrast, our use of high‐quality individually linked data improved the accuracy of the control variables. Using area‐level deprivation scores also runs the risk of reverse causality, where socioeconomic deprivation affects the amount and/or quality of health care received, and vice versa. Our use of education as a time‐invariant measure of socioeconomic status addressed this concern.^28^

Many of the strengths of this study stem from the unique data linkage of rich individual‐level survey data with administrative hospital records. The study was broad in scope (covering the entire NHS hospital system), did not rely on self‐reported measures of hospital use, and distinguished between different types of hospital appointment. This allowed us to comprehensively analyze the nature and scale of educational inequalities in hospital use.

ELSA provided a representative sample of the older adult population in England, which was reweighted to allow for selection into the linked‐ELSA‐HES sample. Focusing solely on participants 65 years and older alleviated concerns about the relationship between work and health, as the vast majority of individuals in the sample were not active in the labor market, and reduced concerns about the differential use of private hospital care across education groups. Individual‐level controls using detailed data were used to adjust for health status to the fullest possible extent.

The data used in this study covered a 10‐year period (2004‐2005 to 2014‐2015) and encompassed a change in the funding regime for the NHS in 2010. We could therefore assess whether educational inequalities in hospital use among older adults changed after 2010, during a time when NHS funding increased at a much slower pace than in the previous decade.

The study has several limitations. First, participants’ use of primary care was not recorded. Previous evidence suggests that lower socioeconomic groups make greater use of primary care than higher socioeconomic groups after controlling for medical needs.^38^ If this continues to be the case, this could offset some of the variation in use of secondary care we observed.

Second, this study, in line with many previous studies, relied on self‐reported measures of health to control for individuals’ need for medical care. Although we supplemented self‐reported data on general health status with data on other self‐reported health complaints and specific diagnoses, these self‐reported health measures alone may not have fully captured an individual's morbidity and need for medical care. Also, membership in a specific socioeconomic group may have influenced how individuals perceived and reported their own health. If low‐education individuals underreported their ill health, individuals from that group would have worse health on average than more educated individuals with the same *observable* level of need. To the extent that this was the case, our estimates of the coefficients on mid‐ and high‐education levels would be biased downward, leading us to understate the extent of any positive education gradient in the use of hospital care. We found, however, that our key conclusions remained unchanged when we excluded the self‐reported health measures most likely to be subject to differential reporting across socioeconomic groups (see Table A2 in the Appendix).

Third, our analysis was based on aggregated national data and therefore did not capture geographical variations in hospital use, which may be an important contributor to overall inequalities in health and health care use.

Fourth, we estimated the extent of educational inequalities in use of *public* health care; we did not observe *private* health care use. A priori, we would expect highly educated people—who have higher incomes on average—to be more likely to have private health insurance or pay for private health care than less‐educated people. Our focus on adults 65 years and older—a population where private insurance rates are considerably lower than among working‐age adults—should mitigate these concerns to some extent; however, some differences in the use of private‐pay options among the education groups might not have been captured. Our estimates therefore likely underestimated the extent of any positive education gradient in *total* health care use.

Finally, we did not directly identify the mechanisms through which differences in hospital use arose. Differences in use of outpatient care across education groups could have been driven by a number of factors, including differences in access to care; differential ability to engage with and adhere to treatment; differences in health care professionals’ behavior toward the various groups; and variation in the tastes and preferences for medical treatment across education groups. This study could not distinguish between these different channels.

## Conclusion

The novel combination of representative survey data on the socioeconomic and health characteristics of older adults in England with administrative hospital records enabled us to study inequalities in hospital use in much greater detail than previously possible. We show that no such educational inequalities exist for both emergency and elective inpatient hospital care. This is an important result. However, we also show that some educational inequalities in the use of hospital care do exist, and that they are growing over time, with less educated respondents using less outpatient care, and more emergency care, than their more educated peers since 2010, after taking into account differences in demographic and health status.

Our analysis was specific to the English setting from 2004 to 2015, and so any policy conclusions drawn for other countries and contexts will come with limitations. Nonetheless, our findings have important implications for US policymakers considering a move toward universal health care. Our evidence suggests that the English system has done a reasonable job of limiting the extent of some, but not all, inequalities in health care use across socioeconomic groups; however, inequalities that do exist increased during a period of tighter funding constraints. Understanding the mechanisms and channels through which this variation arises is essential if effective policy is to be designed to address these inequalities in the United States, United Kingdom, and elsewhere.

*Funding/Support*: George Stoye and Ben Zaranko received support from the Health Foundation (Efficiency Research Programme) and the Economic and Social Research Council (ES/M010147/1). George Stoye and Eric Brunner received support from the British Heart Foundation (RG/16/11/32334).

*Conflict of Interest Disclosures*: All authors complete the ICMJE Form for Disclosure of Potential Conflicts of Interest. No conflicts were reported.

## Supporting information


**Table A1**. Summary Statistics: Self‐Reported Health Conditionsa
**Table A2**. Estimated Relationships Between Education and Use of Outpatient Hospital Services, with and without Adjustment for Demographic and Health Characteristics, 2004‐2005 to 2014‐2015a
**Table A3**. Estimated Relationships Between Education and Use of Outpatient Hospital Services After Adjusting for Demographic and Health Characteristics, by Priority Level, 2004‐2005 to 2014‐2015a
**Table A4**. Estimated Relationships Between Education and Use of Outpatient Hospital Services After Adjusting for Demographic and Health Characteristics, 2004‐2005 to 2008‐2009 vs 2009‐2010 to 2014‐2015a
**Table A5**. Robustness Test: Estimated Relationships Between Education and Use of Hospital Services in the Year After the ELSA Interview, Adjusting for Demographic and Health Characteristics, by Type of Hospital Care, 2004‐2005 to 2014‐2015a
**Table A6**. Robustness Test Using Zero‐Inflated Negative Binomial Model: Incidence Rate Ratios of Relationship Between Education and Use of Hospital Services After Adjusting for Demographic and Health Characteristics, by Type of Hospital Care, 2004‐2005 to 2014‐2015a
**Figure A1**. Relative Hospital Use by Education Group after Adjusting for Demographic Characteristics Only, 2004‐2005 to 2014‐2015a
