# Supplementary material for: Educational Inequalities in Hospital Use Among Older Adults in England, 2004‐2015
Source: Milbank Q. 2020 Oct 6;98(4):1134–70. doi: 10.1111/1468-0009.12479 (PMC7772637; doi:10.1111/1468-0009.12479)
Supplement: Supplementary file 1 — Table A1. Summary Statistics: Self‐Reported Health Conditionsa Table A2. Estimated Relationships Between Education and Use of Outpatient Hospital Services, with and without Adjustment for Demographic and Health Characteristics, 2004‐2005 to 2014‐2015a Table A3. Estimated Relationships Between Education and Use of Outpatient Hospital Services After Adjusting for Demographic and Health Characteristics, by Priority Level, 2004‐2005 to 2014‐2015a Table A4. Estimated Relationships Between Education and Use of Outpatient Hospital Services After Adjusting for Demographic and Health Characteristics, 2004‐2005 to 2008‐2009 vs 2009‐2010 to 2014‐2015a Table A5. Robustness Test: Estimated Relationships Between Education and Use of Hospital Services in the Year After the ELSA Interview, Adjusting for Demographic and Health Characteristics, by Type of Hospital Care, 2004‐2005 to 2014‐2015a Table A6. Robustness Test Using Zero‐Inflated Negative Binomial Model: Incidence Rate Ratios of Relationship Between Education and Use of Hospital Services After Adjusting for Demographic and Health Characteristics, by Type of Hospital Care, 2004‐2005 to 2014‐2015a Figure A1. Relative Hospital Use by Education Group after Adjusting for Demographic Characteristics Only, 2004‐2005 to 2014‐2015a [file MILQ-98-1134-s001.docx]

# **Online Appendix**

**Table A1.** Summary Statistics: Self-Reported Health Conditions^a^

|  | Education level | | |  | |
| --- | --- | --- | --- | --- | --- |
|  | Low | Mid | High | All | |
|  | Mean | Mean | Mean | Mean | SD |
| *Percentage self-reporting ever having been diagnosed with* |  |  |  |  |  |
| Lung disease | 0.12 | 0.09 | 0.05 | 0.09 | 0.29 |
| Asthma | 0.15 | 0.14 | 0.13 | 0.14 | 0.35 |
| Arthritis | 0.54 | 0.47 | 0.43 | 0.49 | 0.50 |
| Osteoporosis | 0.13 | 0.11 | 0.08 | 0.11 | 0.31 |
| Cancer | 0.11 | 0.13 | 0.15 | 0.13 | 0.34 |
| Parkinson’s disease | 0.01 | 0.01 | 0.01 | 0.01 | 0.10 |
| Psychiatric problems | 0.09 | 0.10 | 0.10 | 0.10 | 0.29 |
| Alzheimer’s disease | 0.01 | 0.00 | 0.01 | 0.01 | 0.08 |
| Dementia | 0.03 | 0.01 | 0.01 | 0.02 | 0.13 |
| Blood disorder | 0.01 | 0.01 | 0.01 | 0.01 | 0.08 |
| Hypertension | 0.58 | 0.52 | 0.49 | 0.53 | 0.50 |
| Angina | 0.17 | 0.13 | 0.11 | 0.14 | 0.34 |
| Heart attack | 0.10 | 0.08 | 0.07 | 0.08 | 0.28 |
| Heart failure | 0.01 | 0.02 | 0.01 | 0.01 | 0.11 |
| Heart murmur | 0.07 | 0.06 | 0.07 | 0.07 | 0.25 |
| Heart arrhythmia | 0.12 | 0.14 | 0.16 | 0.14 | 0.34 |
| Diabetes | 0.15 | 0.13 | 0.12 | 0.14 | 0.34 |
| Stroke | 0.10 | 0.06 | 0.06 | 0.07 | 0.26 |
| High cholesterol | 0.40 | 0.42 | 0.40 | 0.41 | 0.49 |
| Observations | 9,611 | 9,688 | 6,565 | 25,864 | |

^a^The sample is restricted to individuals 65 years and older. Mean values are shown for each education group. Education is classified as low (no formal qualifications), mid (completed compulsory education), or high (at least some higher education).

**Table A2.** Estimated Relationships Between Education and Use of Outpatient Hospital Services, with and without Adjustment for Demographic and Health Characteristics, 2004-2005 to 2014-2015^a^

|  | No. of Outpatient Attendances in the Last Year (95% CI) | | | |
| --- | --- | --- | --- | --- |
|  | **Unadjusted** | **Demographic Model^b^** | **Full Model^c^** | **Full Model,  without Selected Self-Reported Health Variables^d^** |
| Panel A: Relative to low-education group | | |  |  |
| Education level: |  |  |  |  |
| Low | 0.0 (Ref) | 0.0 (Ref) | 0.0 (Ref) | 0.0 (Ref) |
| Mid | 0.042  (−0.120 to 0.204) | 0.010  (−0.155 to 0.175) | 0.210  (0.061 to 0.359) | 0.144  (−0.005 to 0.293) |
| High | −0.020  (−0.210 to 0.169) | −0.041  (−0.238 to 0.155) | 0.290  (0.110 to 0.470) | 0.172  (−0.008 to 0.351) |
| Panel B: As a proportion of use in low-education group | | |  |  |
| Mean no. of visits in low-educated group | 2.44 | 2.44 | 2.44 | 2.44 |
| Education level: |  |  |  |  |
| Mid | 1.7%  (−4.9% to 8.4%) | 0.4%  (−6.4% to 7.2%) | 8.6%  (2.5% to 14.7%) | 5.9% (−0.2% to 12.0%) |
| High | −0.8%  (−8.6% to 6.9%) | −1.7%  (−9.8% to 6.4%) | 11.9%  (4.5% to 19.3%) | 7.0% (−0.3% to 14.4%) |

^a^Education is classified as low (no formal qualifications), mid (completed compulsory education), or high (at least some higher education). Data in Panel A show the number of hospital visits for individuals in the high- and mid-education groups relative to those in the low-education group in the year prior to the interview. Data in Panel B show the mean number of hospital visits of each type in the year prior to the interview for those in the low-education group, and the coefficients from Panel A as a proportion of that sample mean. The 95% confidence intervals (CIs) were calculated using robust standard errors clustered at the household level.

^b^Adjusted for English Longitudinal Study of Ageing wave (year), age, age^2^, sex, being nonwhite, being in a couple, being in paid work, and interaction effects between sex and age and sex and being nonwhite.

^c^Additionally adjusted for self-reported general health (fair/poor, good, or very good), scoring ≥4 on the 8-item Centre for Epidemiologic Studies Depression Scale (CES-D) , difficulties with mobility, difficulties with activities of daily living (ADLs), difficulties with instrumental activities of daily living, reporting a long-standing illness, reporting a long-standing and limiting illness, being in receipt of informal long-term care, being in receipt of formal long-term care, whether the individual died in the 2 years following the interview, and whether the individual was ever diagnosed with lung disease, asthma, arthritis, osteoporosis, cancer, Parkinson’s disease, psychiatric problems, Alzheimer’s disease, dementia, a blood disorder, hypertension, angina, heart attack, congestive heart failure, heart murmur, heart arrhythmia, diabetes, stroke, or high cholesterol.

^d^Adjusted as per the full model except for the following exclusions: self-reported general health, CES-D score, reporting a long-standing illness, reporting a long-standing and limiting illness, reporting receipt of informal long-term care and reporting receipt of formal long-term care. Note that other variables (such as ever having been diagnosed with specific ailments, number of difficulties with ADLs, etc) are also self-reported. The variables excluded here are those judged most likely to be subject to biases in subjective reporting between socioeconomic groups.

**Table A3.** Estimated Relationships Between Education and Use of Outpatient Hospital Services After Adjusting for Demographic and Health Characteristics, by Priority Level, 2004-2005 to 2014-2015^a^

|  | Relative No. of Outpatient Encounters in the Last Year (95% CI) | | | | |
| --- | --- | --- | --- | --- | --- |
|  |  | **Priority Type** | | | |
|  | **All** | **Routine** | **Urgent** | **2-Week Referral** | **Unknown** |
| Education level: |  |  |  |  |  |
| Low | 0.0 (Ref) | 0.0 (Ref) | 0.0 (Ref) | 0.0 (Ref) | 0.0 (Ref) |
| Mid | 0.210  (0.061 to 0.359) | 0.202  (0.050 to 0.354) | 0.006  (−0.050 to 0.063) | 0.008  (−0.007 to 0.023) | −0.013  (−0.055 to 0.028) |
| High | 0.290  (0.110 to 0.470) | 0.250  (0.078 to 0.421) | −0.009  (−0.067 to 0.048) | 0.012  (−0.015 to 0.040) | 0.024  (−0.043 to 0.090) |

^a^Education is classified as low (no formal qualifications), mid (completed compulsory education), or high (at least some higher education). Data show the number of outpatient hospital visits for individuals in the high- and mid-education groups relative to those in the low-education group in the year prior to the interview. A 2-week referral is an outpatient appointment made by a general practitioner under the urgent 2-week-wait referral system because a patient’s symptoms might indicate cancer. All data were calculated using the full model, adjusting for English Longitudinal Study of Ageing wave (year) and individual demographic and health characteristics (for full details, see notes to Table A2). The 95% confidence intervals (CIs) were calculated using robust standard errors clustered at the household level.

**Table A4.** Estimated Relationships Between Education and Use of Outpatient Hospital Services After Adjusting for Demographic and Health Characteristics, 2004-2005 to 2008-2009 vs 2009-2010 to 2014-2015^a^

|  | No. of Hospital Visits in the Last Year (95% CI) | | | |
| --- | --- | --- | --- | --- |
|  | **Outpatient** | **Emergency Inpatient** | **Elective Inpatient** | **Emergency Department** |
| 2004-2009^b^ |  |  |  |  |
| Panel A: Relative to low-education group | | |  |  |
| Education level: |  |  |  |  |
| Mid | −0.041  (−0.189 to 0.107) | −0.044  (−0.108 to 0.020) | 0.005  (−0.020 to 0.029) | −0.004  (−0.049 to 0.041) |
| High | 0.136  (−0.074 to 0.346) | −0.036  (−0.112 to 0.040) | −0.013  (−0.038 to 0.013) | 0.013  (−0.042 to 0.068) |
| Panel B: As a proportion of use in the low-education group | | |  |  |
| Mean no. of visits in low-education group | 2.10 | 0.35 | 0.18 | 0.22 |
| Education level: |  |  |  |  |
| Mid | −2.0%  (−9.0% to 5.1%) | −12.6%  (−30.9% to 5.7%) | 2.8%  (−11.1% to 16.1%) | −1.8%  (−22.3% to 18.6%) |
| High | 6.5%  (−3.5% to 16.5%) | −10.3%  (−32.0% to 11.4%) | −7.2%  (−21.1% to 7.2%) | 5.9%  (−19.1% to 30.9%) |
| 2010-2015^c^ |  |  |  |  |
| Panel C: Relative to low-education group | | |  |  |
| Education level: |  |  |  |  |
| Mid | 0.475  (0.245 to 0.704) | 0.024  (−0.048 to 0.096) | −0.013  (−0.048 to 0.023) | −0.011  (−0.056 to 0.033) |
| High | 0.475  (0.211 to 0.740) | 0.023  (−0.055 to 0.101) | −0.015  (−0.043 to 0.014) | −0.037  (−0.075 to 0.001) |
| Panel D: As a proportion of use in the low-education group | | |  |  |
| Mean visits by low educated | 2.82 | 0.42 | 0.22 | 0.33 |
| Education level: |  |  |  |  |
| Mid | 16.8%  (8.7% to 25.0%) | 5.7%  (−11.4% to 22.9%) | −5.9%  (−21.8% to 10.5%) | −3.3%  (−17.0% to 10.0%) |
| High | 16.9%  (7.5% to 26.2%) | 5.5%  (−13.1% to 24.0%) | −6.8%  (−19.5% to 6.3%) | −11.2%  (−22.7% to 0.3%) |

^a^Education is classified as low (no formal qualifications), mid (completed compulsory education), or high (at least some higher education). Data in Panels A and C show the number of hospital visits for individuals in the mid- and high-education groups relative to those in the low-education group in the year prior to the interview, for the 2004-2009 and 2010-2015 periods, respectively. Figures in Panel B and D show the mean number of hospital visits of each type in the year prior to the interview for those with low education, and the coefficients from Panel A as a proportion of that sample mean, for each period. All data were calculated using the full model, adjusting for English Longitudinal Study of Ageing (ELSA) wave (year) and individual demographic and health characteristics (for full details, see notes to Table A2). The 95% confidence intervals (CIs) were calculated using robust standard errors clustered at the household level.

^b^Panels A and B use data from ELSA waves 2, 3, and 4 (2004-2005 to 2008-2009) for outpatient encounters and emergency inpatient and elective inpatient admissions. However, data on emergency department visits were available only from 2008/09 (wave 4) onward.

^c^Panels C and D use data from ELSA waves 5, 6, and 7 (2010-2011 through 2014-2015).

**Table A5.** Robustness Test: Estimated Relationships Between Education and Use of Hospital Services in the Year *After* the ELSA Interview, Adjusting for Demographic and Health Characteristics, by Type of Hospital Care, 2004-2005 to 2014-2015^a^:

|  | No. of Hospital Visits in the Year After Interview  (95% CI) | | | |
| --- | --- | --- | --- | --- |
|  | **Outpatient** | **Emergency Inpatient** | **Elective Inpatient** | **Emergency Department** |
| Education level: |  |  |  |  |
| Mid | 0.260  (0.102 to 0.418) | 0.009  (−0.049 to 0.067) | 0.004  (−0.028 to 0.029) | −0.024  (−0.074 to 0.025) |
| High | 0.252  (0.060 to 0.443) | 0.023  (−0.041 to 0.087) | −0.019  (−0.043 to 0.004) | −0.051  (−0.093 to −0.010) |

^a^Education is classified as low (no formal qualifications), mid (completed compulsory education), or high (at least some higher education). Data show the number of hospital visits for participants in the mid- and high-education groups relative to those in the low-education group in the year after the English Longitudinal Study of Ageing (ELSA) interview. All data were calculated using the full model, adjusting for ELSA wave (year) and individual demographic and health characteristics (for full details, see notes to Table A2). The 95% confidence intervals (CIs) were calculated using robust standard errors clustered at the household level.

**Table A6.** Robustness Test Using Zero-Inflated Negative Binomial Model: Incidence Rate Ratios of Relationship Between Education and Use of Hospital Services After Adjusting for Demographic and Health Characteristics, by Type of Hospital Care, 2004-2005 to 2014-2015^a^

|  | Incidence Rate Ratios for Hospital Use in the Past Year (95% CI) | | | | | | |
| --- | --- | --- | --- | --- | --- | --- | --- |
|  | **Outpatient** | **Emergency Inpatient** | | **Elective Inpatient** | | **Emergency Department** | |
| Education level: |  | |  | |  | |  |
| Mid | 1.046  (0.985 to 1.111) | | 0.985  (0.852 to 1.138) | | 1.005  (0.835 to 1.210) | | 0.984  (0.789 to 1.227) |
| High | 1.103  (1.023 to 1.189) | | 1.019  (0.844 to 1.231) | | 0.946  (0.759 to 1.179) | | 0.870  (0.716 to 1.058) |

^a^Education is classified as low (no formal qualifications), mid (completed compulsory education), or high (at least some higher education). Data show the incidence rate ratio (IRR) for the number of hospital visits for participants in the mid- and high-education groups relative to those in the low-education group in the year prior to the interview. IRRs adjust for English Longitudinal Study of Ageing wave (year) and individual demographic and health characteristics (for full details, see the notes to Table A2). Zero outcomes were inflated using a logit model with the same set of variables. The 95% confidence intervals (CIs) were calculated using robust standard errors clustered at the household level.

**Figure A1.** Relative Hospital Use by Education Group after Adjusting for Demographic Characteristics Only, 2004-2005 to 2014-2015^a^

**
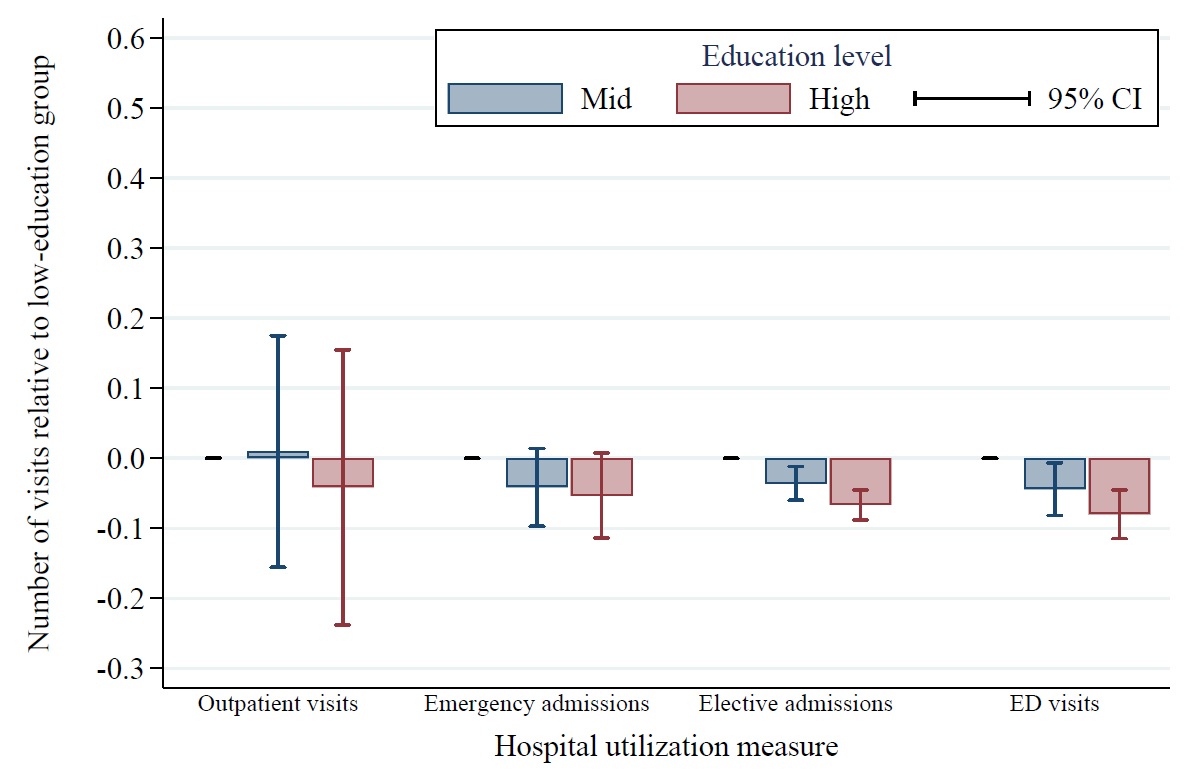
**

^a^Education is classified as low (no formal qualifications), mid (completed compulsory education), or high (at least some higher education). All hospital use measures were calculated relative to use among those in the low-education group, adjusting for English Longitudinal Study of Ageing wave (year) and individual demographic characteristics only (age, age^2^, sex, being nonwhite, being in a couple, being in paid work, and interaction effects between sex and age and sex and being nonwhite). Data on emergency department (ED) visits were available only for 2008-2009 onward.
